# Supplementary figures and images for: Associations between low body mass index and mortality in patients with sepsis: A retrospective analysis of a cohort study in Japan
Source: PLoS One. 2021 Jun 8;16(6):e0252955. doi: 10.1371/journal.pone.0252955 (PMC8186780; doi:10.1371/journal.pone.0252955)

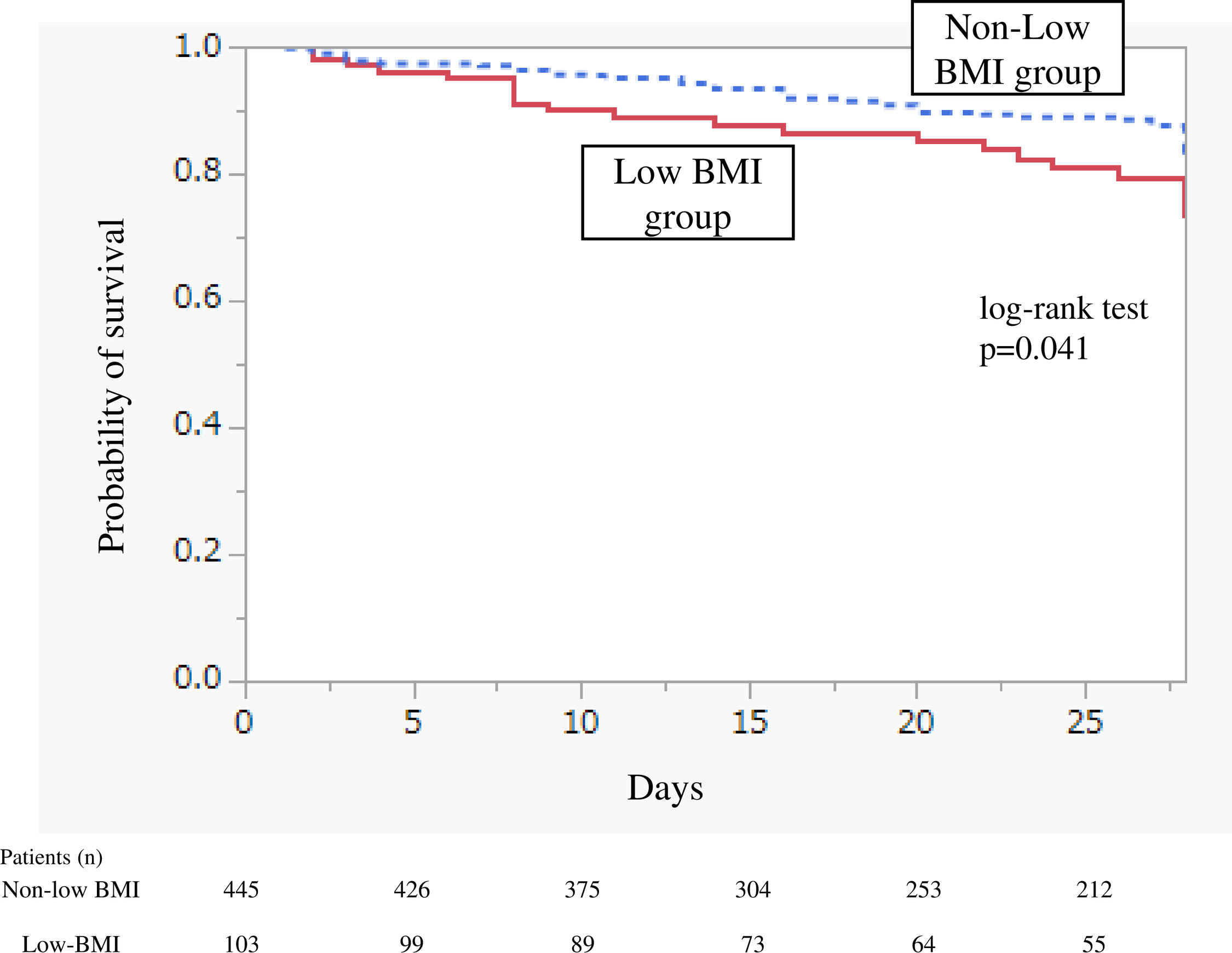

Supplement: S1 Fig — BMI, body mass index. (TIF) [file pone.0252955.s001.tif]
