# Supplementary material for: Associations between low body mass index and mortality in patients with sepsis: A retrospective analysis of a cohort study in Japan
Source: PLoS One. 2021 Jun 8;16(6):e0252955. doi: 10.1371/journal.pone.0252955 (PMC8186780; doi:10.1371/journal.pone.0252955)
Supplement: S2 Table — BMI: body mass index. (DOCX) [file pone.0252955.s003.docx]

**S2 Table.** **Association between BMI and in-hospital mortality in patients with sepsis**

| **Variable** | **OR** | **(95% CI)** | **p-value** |
| --- | --- | --- | --- |
| **Crude** |  |  |  |
| **Low *vs.* normal BMI group** | 1.7 | (0.96–2.8) | 0.068 |
| **High *vs.* normal BMI group** | 1.4 | (0.8–2.3) | 0.197 |
| **Low *vs.* high BMI group** | 1.2 | (0.6–2.2) | 0.59 |
| **Adjusted** |  |  |  |
| **Low *vs.* normal BMI group** | 1.7 | (0.95–3.2) | 0.070 |
| **High *vs.* normal BMI group** | 1.3 | (0.7–2.2) | 0.42 |
| **Low *vs.* high BMI group** | 1.4 | (0.6–2.7) | 0.37 |
| **Age** | 1.0 | (0.9–1.0) | 0.50 |
| **Sex (male vs. female)** | 1.8 | (1.0–3.0) | 0.026 |
| **APACHE Ⅱ scores** | 1.1 | (1.0–1.1) | 0.003 |
| **SOFA scores** | 1.1 | (0.9–1.1) | 0.16 |
| **Shock** | 1.4 | (0.7–2.7) | 0.26 |
| **Pre-existing conditions** | 1.1 | (0.6–1.8) | 0.80 |
| **Lactate level** | 1.0 | (0.9–1.1) | 0.41 |

Abbreviations: APACHE, Acute Physiology and Chronic Health Evaluation; BMI, body mass index; CI, confidence interval; OR, odds ratio; SOFA, Sequential Organ Failure Assessment.
