# Supplementary material for: Associations between low body mass index and mortality in patients with sepsis: A retrospective analysis of a cohort study in Japan
Source: PLoS One. 2021 Jun 8;16(6):e0252955. doi: 10.1371/journal.pone.0252955 (PMC8186780; doi:10.1371/journal.pone.0252955)
Supplement: S3 Table — (DOCX) [file pone.0252955.s004.docx]

**S3 Table. The proportion of missing values in each group**

| Missing values | | |
| --- | --- | --- |
| Variable | Low BMI group  n, (%) | Non-low BMI group  n, (%) |
| Age | 0 | 0 |
| Sex | 0 | 0 |
| APACHE Ⅱ scores | 6 (5.8) | 48 (10.8) |
| SOFA scores | 5 (4.9) | 46 (10.3) |
| Shock | 1 (1) | 6 (1.3) |
| Pre-existing conditions | 0 | 0 |
| Lactate level | 1 (1) | 7 (1.6) |

Abbreviations: APACHE, Acute Physiology and Chronic Health Evaluation; BMI, body mass index; SOFA, Sequential Organ Failure Assessment.

To account for the signiﬁcant proportions of missing values for APACHE II scores (9.9%) and SOFA scores (9.3%), which were assumed to be missing at random, we also conducted our sensitivity analyses with multiple imputation. Multiple imputation through chained equations with predictive mean matching was employed to impute all missing values for the variables and outcomes in the dataset for the logistic regression model. Multiple imputation generated 20 datasets with 20 iterations. As a result, relative to the non-low BMI group, the adjusted OR for 28-day mortality in the low BMI group was 2.3 (95% CI: 1.2–4.2).
