# Supplementary material for: Associations between low body mass index and mortality in patients with sepsis: A retrospective analysis of a cohort study in Japan
Source: PLoS One. 2021 Jun 8;16(6):e0252955. doi: 10.1371/journal.pone.0252955 (PMC8186780; doi:10.1371/journal.pone.0252955)
Supplement: Attachment — Submitted filename: Response_to_Reviewers.docx [file pone.0252955.s005.docx]

March 19, 2021

Dr. Yutaka Kondo

Academic Editor

*PLOS ONE*

U.S. Headquarters

1265 Battery Street Floor 2

San Francisco, CA 94111 United States

Re: Manuscript ID: PONE-D-20-41042

Dear Dr. Kondo and Editors:

Thank you for inviting us to submit a revised draft of our manuscript entitled, “Associations between low body mass index and mortality in patients with sepsis: A retrospective analysis of a cohort study in Japan” to *PLOS ONE*. We also appreciate the time and effort you and each of the reviewers have dedicated to providing insightful feedback on ways to strengthen our manuscript. Thus, it is with great pleasure that we resubmit our manuscript for further consideration. We have incorporated changes that reflect the detailed suggestions you have graciously provided. We also hope that our edits and the responses we have provided below satisfactorily address all the issues and concerns you and the reviewers have noted.

To facilitate your review of our revisions, our point-by-point responses to the questions and comments delivered in your letter dated February 6, 2021 are provided below.

Changes to my financial disclosure: This work was supported by JSPS KAKENHI Grant Number JP19H03755.

Again, thank you for giving us the opportunity to strengthen our manuscript based on your valuable comments and queries. We look forward to hearing from you regarding our submission. We would be glad to respond to any further questions and comments that you may have.

Sincerely,

Tetsuya Sato

Department of Emergency and Critical Care Medicine, Tohoku University Hospital

1-1 Seiryo-machi, Aoba-ku, Sendai 980-8574, Japan

Tel: +8122-717-7489; E-mail: t23jor@med.tohoku.ac.jp

**Responses to Reviewers**

Reviewer #1:

**Q1. You should reconstruct the abstract as a structured abstract (i.e., Background, method, result, conclusion).**

A1. Thank you for your suggestion. We agree that this format would be more appropriate. Accordingly, we have included the required subheadings in the revised Abstract (p. 3, lines 29-49, “Abstract”).

**Q2. In figure 1, you should mention what statistical analysis applied for obtaining the p-value. In addition, you should use "*,** **† or ‡" for showing the significance instead of "a" or "b"**

A2. Thank you for this suggestion. In accordance with your recommendation, we have revised Table 1 to include “†” and “‡” to indicate statistical significance. In addition, the following is included in the legend below the table: “†　and　‡: p values < 0.016 for multiple comparisons (after Bonferroni correction) were considered statistically significant” (p. 8-10, Table 1). A similar statement is included in the Statistical analysis subsection of the Materials and methods (p. 7-8, lines 143-145).

**Q3. Why you chose the logistic model to evaluate 28-days mortality even though Wacharasint et al (Wacharasint et al, Crit Care, 2013) used survival analysis for this issue? Don't you use cox proportional hazard model for this data?**

A3. We thank the reviewer for expressing this concern. As suggested, we utilized a Cox proportional hazards model for survival analysis. The relevant text was added in the Materials and methods (p. 7, lines 131-132) and Results sections (p. 14, lines 205-208, p. 15-16, Table 6). The Kaplan–Meier curves are also shown in S1 Figure (p. 25, lines 388-389).

**Table 6. Cox proportional hazards regression model for 28-day mortality**

| Variable | **HR** | **95% CI** | **p-value** |
| --- | --- | --- | --- |
| Low *vs.* non-low BMI group | 1.7 | 1.0–2.8 | 0.042 |
| Age | 1.0 | 0.9–1.0 | 0.36 |
| Sex (male vs. female) | 1.5 | 0.8–2.5 | 0.14 |
| APACHE Ⅱ scores | 1.0 | 1.0–1.0 | 0.026 |
| SOFA scores | 1.1 | 0.9–1.2 | 0.060 |
| Shock | 0.7 | 0.3–1.4 | 0.34 |
| Pre-existing conditions | 0.9 | 0.5–1.4 | 0.61 |
| Lactate level | 1.1 | 1.0–1.1 | 0.017 |

Abbreviations: APACHE, Acute Physiology and Chronic Health Evaluation; BMI, body mass index; CI, confidence interval; OR, odds ratio; SOFA, Sequential Organ Failure Assessment.

**S1 Figure**

**Q4. You should show the sample size calculation in the method part.**

A4. You have raised an important point; however, unfortunately, this study was a secondary analysis that was not planned in advance, and there was no preset sample size. This point is now described in the Materials and methods section (p. 7, lines 140-141).

**Q5. In table 5, you showed the important result, but I would like to know the whole result of the adjusted model. (age, sex, APACHE II scores, SOFA scores, shock, and pre-existing conditions.)**

A5. Thank you for your suggestion. We agree that the complete results should be included and have thus revised Table 5 in accordance with your recommendation (p. 14-15, Table 5).

**Table 5. Association between BMI and 28-day mortality in patients with sepsis**

| Variable | OR | 95% CI | p-value |
| --- | --- | --- | --- |
| Crude |  |  |  |
| Low *vs.* non-low BMI group | 2.0 | 1.1–3.4 | 0.016 |
| Adjusted^a^ |  |  |  |
| Low *vs.* non-low BMI group | 2.3 | 1.2–4.2 | 0.01 |
| Age | 1.0 | 0.9–1.0 | 0.86 |
| Sex (male vs. female) | 1.7 | 0.9–3.0 | 0.08 |
| APACHE II scores | 1.1 | 1.0–1.1 | 0.006 |
| SOFA scores | 1.1 | 0.9–1.2 | 0.07 |
| Shock | 1.0 | 0.4–2.0 | 0.90 |
| Pre-existing conditions | 1.0 | 0.5–1.8 | 0.95 |
| Lactate level | 1.1 | 0.9–1.1 | 0.15 |

Abbreviations: APACHE, Acute Physiology and Chronic Health Evaluation; BMI, body mass index; CI, confidence interval; OR, odds ratio; SOFA, Sequential Organ Failure Assessment.

**Q6. Page 15, line 208 mistype "eICU".**

A6. Thank you for your comment. We have clarified that “the eICU Collaborative Research Database” refers to a multicenter ICU database that contains high granularity data for over 200,000 admissions to ICUs monitored by eICU Programs across the United States (Philips Healthcare, a major vendor of ICU equipment and services, provides a teleICU service known as the eICU program) (p. 16, lines 227-231).

Reviewer #2:

**Q1. Page 5,** **Section "Materials and methods," subsection "Study design": The authors are requested to briefly describe Tohoku Sepsis Registry, especially regarding how data is collected and whether it is extracted from medical records/billing records/patient surveys.**

A1. Thank you for your suggestion. In accordance with your recommendation, we have revised the manuscript to include the following sentences:

“This study used data from the Tohoku Sepsis Registry (University Hospital Medical Information Network Clinical Trials Registry: UMIN000010297), a multicenter observational cohort study conducted at 10 institutions—including three university hospitals and seven community hospitals—in the Tohoku District in northern Japan.

The detailed design of this study has been reported previously [22]. Briefly, the Tohoku Sepsis Registry prospectively enrolled consecutive patients admitted to an intensive care unit (ICU) with severe sepsis or patients who developed severe sepsis after admission to an ICU between January 2015 and December 2015. Severe sepsis and septic shock were defined according to the 2012 International Sepsis Guidelines [23]. Patients aged <18 years were excluded from the registry. Researchers at each institution collected data from patient medical records and registered them in the web registration system.” (p. 5-6, lines 92-102).

**Q2. Page 6, Section "Materials and methods," subsection "Definitions and outcome measures": The authors have categorized BMI into low, normal, and high based on Japanese Guidelines for Management of Obesity Disease. Did the authors consider keeping BMI as a continuous variable and analyzing the association of BMI (as a continuous variable) with 28-day mortality and the secondary outcomes (all-cause in-hospital mortality and ICU-free days)?**

A2. Thank you for your suggestion. You have raised an important question. However, continuous variables are commonly used when the relationship between variables and outcomes are predicted to exhibit linear regression. We have searched the literature for previous studies regarding whether linear or non-linear regression is predicted for the relationship between BMI and the outcome investigated in the present study. Based on the reports by Zhang et al. [1], Sakr et al. [2], and Niedziela et al. [3], non-linear regression was predicted for the relationship between BMI and mortality (please see the figures in each of the references below). Thus, in our case, we judged it appropriate to use BMI as a categorical variable. In fact, the crude 28-day mortality rates in this study were 21.4% in the low BMI group, 11.2% in the normal BMI group, and 14.5% in the high BMI group, indicating a U-shaped relationship between BMI and mortality (i.e., non-linear regression). The same tendency was observed for crude in-hospital mortality, although the relationship between BMI and ICU-free days exhibited a reversed U-shape.

1. Zheng, W., et al. Association between body-mass index and risk of death in more than 1 million Asians. N Engl J Med. 2011; 364(8): 719-729.

2. Sakr, Y., et al. Being overweight or obese is associated with decreased mortality in critically ill patients: a retrospective analysis of a large regional Italian multicenter cohort. J Crit Care. 2012; 27(6): 714-721.

3. Niedziela, J., et al. The obesity paradox in acute coronary syndrome: a meta-analysis. Eur J Epidemiol. 2014; **29**(11): 801-812.

**Q3. Page 6, Section "Materials and methods," subsection "Definitions and outcome measures": The authors are requested to describe how the 28-day mortality was measured? Were the patients followed up after discharge? Was the mortality all-cause mortality?**

A3. Thank you for your suggestion. As indicated, we have revised the text as follows: “The primary outcome measure was all-cause 28-day mortality. No follow-up after discharge, survival discharge, or survival during hospitalization within 28 days was regarded as survival, and in-hospital death within 28 days was regarded as death.” (p. 6-7, lines 118-120).

**Q4. Page 6, Section "Materials and methods," subsection "Statistical analyses":**

**Q4-1. The authors mention that the dataset had missing values. The authors are requested to describe how much of the data was missing and whether it affected the results from the analyses.**

A4-1. Thank you for your suggestion. In accordance with your recommendation, we have described the missing values and sensitivity analyses in S3 Table (p. 14, lines 208-209, p. 25, line 394). In addition, to account for the signiﬁcant proportions of missing values for APACHE II (9.9%) and SOFA scores (9.3%) that were assumed to be missing at random, we conducted our sensitivity analyses with multiple imputation. Multiple imputation through chained equations with predictive mean matching was employed to impute all missing values for the variables and outcomes in the dataset for the logistic regression model. Multiple imputation generated 20 datasets with 20 iterations. As a result, relative to the non-low BMI group, the adjusted OR for 28-day mortality in the low BMI group was 2.3 (95% CI: 1.2–4.2) (S3 Table). This result was consistent with the results of the analysis of the original data.

**S3 Table. The proportion of missing values in each group**

| Variable | **Low BMI group**  **n, (%)** | **Non-low BMI group**  **n, (%)** |
| --- | --- | --- |
| Age | 0 | 0 |
| Sex | 0 | 0 |
| APACHE Ⅱ scores | 6 (5.8) | 48 (10.8) |
| SOFA scores | 5 (4.9) | 46 (10.3) |
| Shock | 1 (1) | 6 (1.3) |
| Pre-existing conditions | 0 | 0 |
| Lactate | 1 (1) | 7 (1.6) |

Abbreviations: APACHE, Acute Physiology and Chronic Health Evaluation; BMI, body mass index; SOFA, Sequential Organ Failure Assessment.

**Q4-2. How were the pre-existing conditions identified? Were they extracted from** **medical records or billing codes, or patient interviews?**

A4-2. Thank you for these relevant questions. Please see A1 above. The revised text now indicates that researchers at each institution retrieved the data regarding pre-existing conditions from patient medical records (p. 6, lines 101-102).

**Q4-3. The authors are requested whether they considered controlling the multivariable logistic regression for serum lactate levels? It is known that reduction in lactate level is associated with survival from sepsis. Hence including this variable in the model appears to be necessary.**

A4-3. Thank you for your suggestion. We agree that this information is necessary and have incorporated data for lactate levels in the text and tables (p. 7, line 136, p. 14-16, Tables 5 and 6).

**Table 5. Association between BMI and 28-day mortality in patients with sepsis**

| **Variable** | **OR** | **95% CI** | **p-value** |
| --- | --- | --- | --- |
| **Crude** |  |  |  |
| **Low vs. non-low BMI group** | 2.0 | 1.1–3.4 | 0.016 |
| **Adjusted** |  |  |  |
| **Low vs. non-low BMI group** | 2.3 | 1.2–4.2 | 0.01 |
| **Age** | 1.0 | 0.9–1.0 | 0.86 |
| **Sex (male vs. female)** | 1.7 | 0.9–3.0 | 0.08 |
| **APACHE II scores** | 1.1 | 1.0–1.1 | 0.006 |
| **SOFA scores** | 1.1 | 0.9–1.2 | 0.07 |
| **Shock** | 1.0 | 0.4–2.0 | 0.90 |
| **Pre-existing conditions** | 1.0 | 0.5–1.8 | 0.95 |
| **Lactate level** | 1.1 | 0.9–1.1 | 0.15 |

Abbreviations: APACHE, Acute Physiology and Chronic Health Evaluation; BMI, body mass index; CI, confidence interval; OR, odds ratio; SOFA, Sequential Organ Failure Assessment.

**Table 6. Cox proportional hazards regression model for 28-day mortality**

| Variable | HR | 95% CI | p-value |
| --- | --- | --- | --- |
| Low *vs.* non-low BMI group | 1.7 | 1.0–2.8 | 0.042 |
| Age | 1.0 | 0.9–1.0 | 0.36 |
| Sex (male vs. female) | 1.5 | 0.8–2.5 | 0.14 |
| APACHE Ⅱ scores | 1.0 | 1.0–1.0 | 0.026 |
| SOFA scores | 1.1 | 0.9–1.2 | 0.060 |
| Shock | 0.7 | 0.3–1.4 | 0.34 |
| Pre-existing conditions | 0.9 | 0.5–1.4 | 0.61 |
| Lactate | 1.1 | 1.0–1.1 | 0.017 |

Abbreviations: APACHE, Acute Physiology and Chronic Health Evaluation; BMI, body mass index; CI, confidence interval; OR, odds ratio; SOFA, Sequential Organ Failure Assessment.

**Q4-4. The authors are requested whether they considered controlling the multivariable logistic regression for other laboratory values, medications, socioeconomic status, etc.?**

A4-4. Thank you for your suggestion. You have raised an important point; however, there was a limit to the number of variables that could be adopted based on the number of outcomes, and variables considered to be clinically more important were adopted. Laboratory values and medications are also important, but they are judged to have low priority. To address this issue, we have revised the text as follows: “We deemed these variables potentially important based on clinical judgment and past sepsis research [27]. A previous study also indicated that body weight may be associated with age and pre-existing conditions [28].” (p. 7, lines 136-138). There were no data related to socioeconomic status. Please also see A7 below.

**Q5. Page 12, Section "Results," subsection "Associations between BMI and outcomes": The authors are requested to rephrase or remove** **the following sentence “Although there were no significant differences, patients in the low BMI group had a higher odds ratio…..”. The confidence intervals for the odds ratios mentioned in this sentence are wide; hence it will give the readers a false impression that low BMI had a higher odds ratio for 28-day mortality than high BMI.**

A5. Thank you for this suggestion. As indicated, we have deleted the following sentence: “Although there were no significant differences, patients in the low BMI group had a higher odds ratio…” (p. 12, line 186).

**Q6. Table 5, S1 Table, S2 Table: The authors are requested to provide the odds ratio, 95% CI, and p-values for all the variables (such as age, sex, APACHE II scores, etc.) in the regression model.**

A6. Thank you for this suggestion. We agree that information regarding these variables should be included in the tables. Accordingly, we have incorporated your suggestion in Table 5 (p. 14-15), S1 Table, and S2 Table (p. 25, lines 390-393).

**Table 5** (see above)

**S1 Table. Association between BMI and 28-day mortality in patients with sepsis**

| **Variable** | **OR** | **(95% CI)** | **p-value** |
| --- | --- | --- | --- |
| **Crude** |  |  |  |
| **Low vs. normal BMI group** | 2.2 | (1.2–3.9) | 0.010 |
| **High vs. normal BMI group** | 1.4 | (0.7–2.5) | 0.32 |
| **Low vs. high BMI group** | 1.6 | (0.8–3.2) | 0.173 |
| **Adjusted** |  |  |  |
| **Low vs. normal BMI group** | 2.4 | (1.2–4.7) | 0.009 |
| **High vs. normal BMI group** | 1.3 | (0.6–2.5) | 0.53 |
| **Low vs. high BMI group** | 1.9 | (0.9–4.3) | 0.094 |
| **Age** | 1.0 | (0.9–1.0) | 0.83 |
| **Sex (male vs. female)** | 1.7 | (0.9–3.0) | 0.09 |
| **APACHE II scores** | 1.1 | (1.0–1.1) | 0.006 |
| **SOFA scores** | 1.1 | (0.9–1.2) | 0.07 |
| **Shock** | 0.9 | (0.4–1.9) | 0.85 |
| **Pre-existing conditions** | 1.0 | (0.5–1.8) | 0.98 |
| **Lactate level** | 1.1 | (0.9–1.1) | 0.14 |

Abbreviations: APACHE, Acute Physiology and Chronic Health Evaluation; BMI, body mass index; CI, confidence interval; OR, odds ratio; SOFA, Sequential Organ Failure Assessment.

**Table S2. Association between BMI and in-hospital mortality in patients with sepsis**

| **Variable** | **OR** | **(95% CI)** | **p-value** |
| --- | --- | --- | --- |
| **Crude** |  |  |  |
| **Low vs. normal BMI group** | 1.7 | (0.96–2.8) | 0.068 |
| **High vs. normal BMI group** | 1.4 | (0.8–2.3) | 0.197 |
| **Low vs. high BMI group** | 1.2 | (0.6–2.2) | 0.59 |
| **Adjusted** |  |  |  |
| **Low vs. normal BMI group** | 1.7 | (0.95–3.2) | 0.070 |
| **High vs. normal BMI group** | 1.3 | (0.7–2.2) | 0.42 |
| **Low vs. high BMI group** | 1.4 | (0.6–2.7) | 0.37 |
| **Age** | 1.0 | (0.9–1.0) | 0.50 |
| **Sex (male vs. female)** | 1.8 | (1.0–3.0) | 0.026 |
| **APACHE II scores** | 1.1 | (1.0–1.1) | 0.003 |
| **SOFA scores** | 1.1 | (0.9–1.1) | 0.16 |
| **Shock** | 1.4 | (0.7–2.7) | 0.26 |
| **Pre-existing conditions** | 1.1 | (0.6–1.8) | 0.80 |
| **Lactate level** | 1.0 | (0.9–1.1) | 0.41 |

**Q7. Page 16, Section "Discussion": The authors are requested to explain what they mean by “confounders used for adjustment in the multivariate analysis were limited because the number of patients and non-survivors was not large enough.”**

A7. Thank you for your suggestion. We have replaced the term [confounders] throughout the paper with [variables] to use more precise terms. We have also revised the text to include the following: “Given that there were 76 non-survivors based on the number of event occurrences, we used eight adjustment variables to develop a suitable statistical model.” (p. 18, lines 265-267).

**Figures and Tables:**

To improve the manuscript based on the reviewers’ comments, we have made the following changes to the figures and tables:

A new figure has been inserted as S1 Figure in the Supporting Information.

Tables 1, 3, and 5 have been revised.

A new table has been inserted as Table 6 in the manuscript.

A new table has been inserted as S3 Table in the Supporting Information.
