# Supplementary material for: Associations between low body mass index and mortality in patients with sepsis: A retrospective analysis of a cohort study in Japan
Source: PLoS One. 2021 Jun 8;16(6):e0252955. doi: 10.1371/journal.pone.0252955 (PMC8186780; doi:10.1371/journal.pone.0252955)
Supplement: Attachment — Submitted filename: Response_to_Reviewers.docx [file pone.0252955.s006.docx]

April 26, 2021

Dr. Yutaka Kondo

Academic Editor

*PLOS ONE*

U.S. Headquarters

1265 Battery Street Floor 2

San Francisco, CA 94111 United States

Re: Manuscript ID: PONE-D-20-41042R1

Dear Dr. Kondo and Editors:

Thank you for your email dated April 14, 2021 enclosing the reviewers’ comments for our manuscript entitled, “Associations between low body mass index and mortality in patients with sepsis: A retrospective analysis of a cohort study in Japan”. We have carefully reviewed the comments and have revised the manuscript accordingly. Our responses are given in a point-by-point manner below.

We hope the revised version is now suitable for publication and look forward to hearing from you in due course. We would be glad to respond to any further questions and comments that you may have.

Sincerely,

Tetsuya Sato

Department of Emergency and Critical Care Medicine, Tohoku University Hospital

1-1 Seiryo-machi, Aoba-ku, Sendai 980-8574, Japan

Tel: +8122-717-7489; E-mail: [t23jor@med.tohoku.ac.jp](mailto:t23jor@med.tohoku.ac.jp), t23jor@gmail.com

**Responses to Reviewers**

Reviewer #1: Thank you for your review of our paper.

Reviewer #2: Thank you for your comments. Our answers to your points are as follows.

**Q1. Section ‘Material and Methods’: Since the authors have conducted survival analysis, the authors are requested to define how survival was measured. The authors have already provided an explanation for how they measured all-cause 28-day mortality. The authors are requested to add a sentence in this context on how survival was measured such as** **“survival was measured as number of days from admit date to death” etc. Also, the authors are requested to add a sentence on how patients were censored.**

A1. Thank you for your suggestion. In accordance with your recommendation, we have revised the manuscript to include the following sentences:

“In survival time analysis, survival was measured as number of days from admit date to death and censor was defined as survival discharge within 28 days” (p. 7, lines 120-122).

**Q2. Table 1: Few cells in this table have superscripted ‘b’ instead of † or ‡. The authors are requested to correct them.**

A2. Thank you for your point-out, we have corrected Table 1. We have changed ‘b’ to ‘‡’ (p. 9-10, Table 1).
